# The seroprevalence of *Mycoplasma pneumoniae* IgM and IgG antibodies in patients with ischemic stroke

**Published:** 2016-12

**Authors:** Maryam Roham, Khatereh Anbari, Samira Mirhabibi, Gholamreza Goudarzi

**Affiliations:** 1 Antimicrobial-Resistant Research Center, Iran University of Medical Sciences, Motahari Burn Hospital, Tehran, Iran; 2 Faculty of Medicine, Lorestan University of Medical Sciences, Khoramabad, Iran

**Keywords:** *Mycoplasma pneumoniae*, CVA, Iran, Seroprevalence

## Abstract

**Background and Objectives:**

Association between *Mycoplasma pneumoniae* infection and increased risk for brain stroke has been well understood. Hence, the value of serologic tests for assessing causative relationship between this infection and brain stroke seems to be high. The present study aimed to determine serum level of anti-*Mycoplasma pneumoniae* antibodies in patients with brain stroke and to compare it with non-stroke patients.

**Materials and Methods:**

This cross-sectional study was performed on 97 consecutive ischemic stroke patients and 97 sex and age-matched non-stroke patients. Quantitative enzyme-linked immunosorbent assay (ELISA) was established to measure the levels of anti-*Mycoplasma pneumoniae* IgG and IgM antibodies.

**Results:**

Regarding the level of anti-*Mycoplasma pneumoniae* IgM, the titer of this marker was positive in 4.1% of patients with ischemic stroke, while none of the subjects in control group had positive titer for this antibody (OR = 1.043, 95%CI: 1.001 – 1.087, p = 0.043). The rate of positivity for anti-*Mycoplasma pneumoniae* IgG in ischemic stroke patients was significantly higher than in the control group (28.5% versus 13.4%, p = 0.031). Odds ratio for exposure to *M. pneumoniae* was 2.24 times of the control subjects. The level of anti-*Mycoplasma pneumoniae* IgM was independent to both sex and age variables in patients group (p = 0.77). The level of anti-*Mycoplasma pneumoniae* IgG did not depend on subjects’ gender in control group, but was significantly higher in men compared with women in patients group.

**Conclusion:**

A high level of anti-*Mycoplasma pneumoniae* IgM and IgG antibodies indicate a significant association of *M. pneumoniae* infection and history of this infection with increased risk for ischemic stroke.

## INTRODUCTION

One of the possible risk factors causing stroke has considered to be infections (1, 2). *Chlamydophyla pneumoniae*, is one of the respiratory pathogen which has been connected to atherosclerotic vascular diseases (3, 4). *Mycoplasma pneumoniae*, is another micro-organism which has the same epidemiological behaviour that after respiratory tract infection can bring extra pulmonary symptoms and chronic sequelae (5, 6).

There has been growing evidence showing that in spite of the main presentation of *M. pneumoniae* infection which cause respiratory problems, it also has the ability in producing a wide range of extra pulmonary manifestations including neurologic, cardiac, dermatologic, musculoskeletal, hematologic, and gastrointestinal (7).

The most common and life-threatening extrapulmonary complications of *Mycoplasma pneumoniae* infection are central nervous system (CNS) manifestations (8, 9). The prevalence of *M. pneumoniae*-associated neurologic complications is not clear. Among patients with neurologic syndromes, *M. pneumoniae* association has been shown in 5% to 10% of cases (10, 11).

Various epidemiological, serological, and even genetic studies could show an association between *M. pneumoniae* infection and increased risk for neurological complications (12–16). Even, exposure to *M. pneumoniae* infection has been shown to adversely affect prognosis of these affected patients (17). According to some reports, neurological defects may occur in 0.1% of *M. pneumoniae* infections that is mostly manifested by neurological infections or inflammatory conditions such as meningitis, encephalitis, cerebellar syndromes, myelitis, and polyradiculitis (18). However, an unusual manifestation of *M. pneumoniae* infection is brain stroke due to the potential impact of this infection on occurring vasculopathies (19). Because the clinical symptoms and imaging evidences of brain stroke due to *M. pneumoniae* infection is not specific, the use of more accurate diagnostic test can help the clinicians to early diagnosis of stroke caused by this infection and lead to proper selection of the best therapeutic modality against this infection (20). In this regard, the serological test is one way to detect the origin of infection in its acute or chronic phases of infection.In fact, markedly elevation of serum antibody titers to *M. pneumoniae* and the detection of specific IgM and IgG antibodies against this microorganism can be one of the serological diagnostic choice to confirm brain stroke specifically caused by *Mycoplasma pneumoniae* (21). Furthermore, applying specific serological test along with genetically assessing the presence of *M. pneumoniae* DNA may result in obtaining the highest accuracy for detecting infection in brain stroke patients (22). Our case-control study was the prospectively to examine for the seroprevalence of *M. pneumoniae*, and its relationship with acute stroke.

## MATERIALS AND METHODS

### Study population

This epidemiological cross-sectional study was performed on 97 consecutive patients aged 45 to 80 years referred to Shohada-e-Ashaayer hospital in Khorramabad, Iran between August 2012 to August 2013 with clinical manifestations and ischemic lesions in brain CT that finally diagnosed as ischemic cerebrovascular accidents. Those with rheumatologic or inflammatory disorders such as lupus, scleroderma, Sjogren’s syndrome or vasculitis were not included into this survey. Also, 97 sex, and age-matched subjects without any evidences of cerebrovascular diseases were selected from hospitalized patients in internal wards or outpatients referred to laboratory of hospital as the control. Baseline characteristics and clinical data of study participants were collected by face to face interviewing in the presence the patient’s bedside.

### Study measurements

The results of laboratory parameters including fasting blood sugar and lipid profile as well as blood pressure measures were also collected from recorded hospital files. For all patients fasting blood sugar, cholesterol, triglyceride, Low density lipoprotein and High density lipoprotein measured. After the implementation and control of the CT images and taking informed consent to participate in the project, 0.5 ml of serum was extracted, centrifuged, and transferred into micro-tubes that were kept frozen at −20°C until antibodies determination. Quantitative enzyme-linked immunosorbent assays (ELISAs) were established to measure the levels of anti-*M. pneumoniae* IgG and IgM antibodies using *M. pneumoniae* IgG ElISA and *M. pneumoniae* IgM ElISA kits (IBL, Germany). The assay was considered positive if the level of IgM and IgG ≥ 1.1 mg and was considered borderline if the level of antibodies ranged 0.8 to 1.1 mg. For borderline samples, the test was repeated and if the titer was ranged in the borderline ranges, it was considered as negative.

### Statistical analysis

Results were presented as mean ± standard deviation (SD) for quantitative variables and were summarized by absolute frequencies and percentages for categorical variables. Categorical variables were compared using chi-square test or Fisher’s exact test when more than 20% of cells with expected count of less than 5 were observed. Quantitative variables were also compared with Mann-Whitney U test. Statistical significance was determined as a p value of ≤ 0.05. All statistical analysis was performed using SPSS software (version 19.0, SPSS Inc., Chicago, Illinois).

## RESULTS

Overall, 97 patients with ischemic stroke and 97 healthy controls were assessed with respect to the levels of anti-*M. pneumoniae* antibodies. The two groups were matched for mean age (69.1 ± 9.3 years versus 67.2 ± 8.9 years, p = 0.15), female gender distribution (62.9% versus 52.6%, p = 0.14), and also occupational state (Table 1). There was also no difference in the prevalence rates of cardiovascular risk factors between the patients and the control including hyperlipidemia (8.3% versus 6.8%, p = 0.446), hypertension (32.4% versus 34.5%, p = 779), diabetes mellitus (5.4% versus 7.1%, p = 0.06), and also in combination of these risk profiles (Table 1). Also, the mean serum levels of biomarkers including fasting blood sugar, and lipid profile were similar in both study groups.

**Table 1.** Baseline characteristics and clinical data of patients and controls

**Characteristics**		**Patients group (n = 97)**	**Control group (n = 97)**	**P-value**
**Gender**	**Male**	36 (37.1)	46 (47.4)	0.14
	**Female**	61 (62.9)	51 (52.6)	
**Mean age, year**		69.1 ± 9.3	67.2 ± 8.9	0.15
**Age group**	**45 – 54 years**	7 (7.2)	12 (12.4)	
	**55 – 69 years**	36 (37.1)	37 (38.1)	0.43
	**≥ 70 years**	54 (55.7)	48 (49.5)	
**Medical history**	**Hyperlipidemia**	8 (8.3)	6 (6.8)	0.60
	**Hypertension**	31 (32.4)	33 (34.5)	0.82
	**Diabetes mellitus**	5 (5.4)	7 (7.1)	0.57
	**Hyperlipidemia+hypertension**	17 (17.5)	21 (21.4)	0.55
	**Diabetes + hypertension**	9 (9.5)	9 (9.5)	1.00
	**Hyperlipidemia + hypertension + diabetes**	17 (17.5)	15 (15.5)	0.74
	**Hypertension + heart failure**	10 (10.8)	3 (3.6)	0.06
**Occupational status**	**Unemployed**	27 (27.8)	29 (29.9)	0.81
	**Housekeeper**	50 (50.5)	48 (48.5)	0.86
	**Farmer**	8 (8.2)	6 (6.2)	0.60
	**Worker**	4 (4.1)	9 (9.3)	0.17
	**Retired**	2 (2.1)	0 (0.0)	0.49
	**Employed**	6 (6.2)	7 (7.2)	0.78
**Laboratory indices**	**Fasting blood sugar, mg/dl**	123.23 ± 56.5	116.54 ± 37.8	0.43
	**Total cholesterol, mg/dl**	197.5 ± 55.1	189.1 ± 61.1	0.33
	**Triglyceride, mg/dl**	169.78 ± 68.2	156.36 ± 47.5	0.44
	**Low density lipoprotein, mg/dl**	105.5 ± 36.5	117.13 ± 77.7	0.32
	**High density lipoprotein, mg/dl**	54.2 ± 33.4	51.1 ± 27.2	0.68

Regarding the level of anti-*Mycoplasma pneumoniae* IgM, the titer of this marker was positive in 4.1% of patients with ischemic stroke, while none of the subjects in control group had positive titer for this antibody (OR = 1.043, 95%CI: 1.001 – 1.087, p = 0.043). Moreover, the rate of positivity for anti-*Mycoplasma pneumoniae* IgG in ischemic stroke patients was significantly higher than in the control group (28.5% versus 13.4%, p = 0.031). In this regard, odds ratio for exposure to *M. pneumoniae* was 2.24 times of the control subjects (OR = 2.24, 95%CI: 1.070 – 4.700, P = 0.031) (Table 2). The level of anti-*Mycoplasma pneumoniae* IgM was independent to both sex and age variables in patients group that the titer for IgM antibody was positive in none of the patients in the age range 45 to 54 years, in 5.6% of patients in the age range 55 to 69 years and in 3.7% of patients older than 69 years (p = 77). Also, 8.3% of affected men and 1.6% of affected women had positive titer for anti-*Mycoplasma pneumoniae* IgM (p = 0.14). As shown in Table 3, the level of anti-*Mycoplasma pneumoniae* IgG did not depend on subjects’ gender in control group, but was significantly higher in men compared with women in patients group. This table also shows that the level of anti-*Mycoplasma pneumoniae* IgG is independent to age distribution in both patients and controls groups.

**Table 2.** Seroprevalence of *M. pneumoniae* antibody in cases and controls

**Anti-Mycoplasma antibodies**	**Groups**	**Positive (%)**	**Negative (%)**	**P. value**	**OR**	**CI**
**Ig M**	Patients (sign of ischemia in brain CT scan)	4(4.1)	93(95.9)	0.043	1.043	1.001–1.087
	Control group	0(0)	97(100)			
**Ig G**	Patients (sign of ischemia in brain CT scan)	25(28.1)	72(74.2)	0.031	2.24	1.070–4.700
	Control group	13(13.4)	84(86.6)			

**Table 3.** The positive titer for the level of anti-*Mycoplasma pneumoniae* IgG

**Characteristics**		**Patients group (n = 97)**	**Control group (n = 97)**
**Gender**	**Male**	13 (36.1)	6 (13.0)
	**Female**	12 (19.7)	7 (13.7)
**p-value**		0.047	0.92
**Age groups**	**45 – 54 years**	2 (28.6)	2 (16.7)
	**55 – 69 years**	7 (19.4)	5 (13.5)
	**≥ 70 years**	16 (29.6)	6 (12.5)
**p-value**		0.54	0.94

## DISCUSSION

Because of important role of *M. pneumoniae* in cerebrovascular complications measuring of antibodies in serum or cerebrospinal fluid is highly valuable in diagnosis of this infection and its related neurological complications. The presents study found a significant difference in the serum level of both anti-*Mycoplasma pneumoniae* IgM and IgG antibodies between the patients with brain stroke and non-stroke individuals who referred to hospital because of other complaints. In this study, odds ratio for exposure to *M. pneumoniae* was 2.24 times in brain stroke group than in the control subjects indicating an increased risk for occurrence of ischemic brain stroke and *M. pneumoniae.* Because the levels of both types of acute and chronic phase antibodies were higher in patients group, it seems that the risk for brain stroke in those patients with the previous history of *M. pneumoniae* infection can be even increased. In our study, difference in the seroprevalence rate of antibodies between the patients and the controls was significant (p=0.031 and p = 0.043 for IgG and IgM).

In the study of Ngeh and colleagues, the seroprevalence of *M. pneumoniae* IgG in the stroke were 61% that was considerably higher than in our survey. Using a logistic regression statistical model, adjusting for cardiovascular risk factors, the odds ratios of having a stroke or in relation to *M. pneumoniae* IgG in their study was 1.32 which is lower than that obtained in our study. One of the main reasons for explaining the contradictory results of the present study and above-mentioned study may be the different employed age subgroups so Ngeh and colleagues focused on the older population (23). In another study conducted by Min et al. the serum level of *M. pneumoniae* acute phase antibody was significantly increased simultaneously with the appearance of neurological manifestations in the patients referred with hemiparesis and facial nerve paralysis. In their study, the level of antibody increased in both serum and cerebrospinal fluid. According to this fact that the changes in antimicrobial antibodies in cerebrospinal fluid can be more specific than in the serum, the measurement of acute and chronic phase antibodies in cerebrospinal fluid leading higher diagnostic value (24).

In another study by Chiang CH et al. it was found that subjects with *M. pneumoniae* infection were significantly associated with increased risk of ischemic stroke compared with controls (1.10% versus 0.72%, respectively; P 0.01) (19).

Another important result in our study was that elevation of anti-*Mycoplasma pneumoniae* antibody is independent to patients’ age; however this elevation may be more occurred in men than in women emphasizing higher risk for cerebrovascular accidents following *M. pneumoniae* infection in men than in women. Also, numerically, but not significantly, the rate of seropositivity was higher in advanced ages. Tuuminen et al. could show an association between the rate of seropositivity and patients’ age in those who suffered brain stroke (25). In another study Taghizade et al. the relationship between seropositivity rate *M. pneumoniae* antibodies and gender in patient with acute myocardial infarction remained insignificant (26). It seems that the higher rate of brain stroke caused by *M. pneumoniae* infection in men than in women may be due to higher socially and occupationally exposing this infection in men compared with women. In this regard, the gender difference in the rate of brain stroke related to *M. pneumoniae* infection should be more assessed in larger population-based studies.

Other effects of *Mycoplasma* can be seen in researches conducted in another articles. Among these we can mention two inter-related articles conducted by Golmohamadi and Ataee. They concluded that *M. pneumoniae*, *Mycoplasma hominis,* and *Mycoplasma arthritidis* have increased in RA patients (27, 28). Another study carried out on women in Albania with infertility and abortion showed another effect of *Mycoplasma hominis* (29).

In conclusion, a higher level of anti-*Mycoplasma pneumoniae* acute and chronic phase antibodies is detectable in patients with ischemic stroke than in non-stroke patients. On the other hand, high level of anti-*Mycoplasma pneumoniae* IgM and IgG antibodies indicate a significant association between *M. pneumoniae* infection and history of this infection and increased risk for ischemic stroke.

According to the results that obtained in recent studies clinicians should be aware of this potential association between *M. pneumoniae* infection and several CNS manifestations, when confronted with nervous symptoms of unknown cause especially if the patient’s history includes respiratory manifestation.

They should attempt to establish this association whenever possible with the aid of molecular or serological techniques. Further studies may be required to clarify and define the meticulous role of *M. pneumoniae* infection as a potential factor in the pathogenesis of ischemic stroke, in all age groups, and in different race populations. A better understanding of the pathogenesis of such manifestations will not only help the clinician diagnose and treat this rare entity but may also lead to new insight into complex neurological injury and its potential association with infectious agents.
